# The N-Terminal Domains of Vps3 and Vps8 Are Critical for Localization and Function of the CORVET Tethering Complex on Endosomes

**DOI:** 10.1371/journal.pone.0067307

**Published:** 2013-06-20

**Authors:** Nadine Epp, Christian Ungermann

**Affiliations:** Biochemistry Section, Department of Biology/Chemistry, University of Osnabrück, Osnabrück, Germany; University of Geveva, Switzerland

## Abstract

Endosomal biogenesis depends on multiple fusion and fission events. For fusion, the heterohexameric CORVET complex as an effector of the endosomal Rab5/Vps21 GTPase has a central function in the initial tethering event. Here, we show that the CORVET-specific Vps3 and Vps8 subunits, which interact with Rab5/Vps21, require their N-terminal domains for localization and function. Surprisingly, CORVET may lack either one of the two N-terminal domains, but not both, to promote protein sorting via the endosome. The dually truncated complex mislocalizes to the cytosol and is impaired in endocytic protein sorting, but not in assembly. Furthermore, the endosomal localization can be rescued by overexpression of Vps21 or one of the truncated CORVET subunits, even though CORVET assembly is not impaired by loss of the N-terminal domains or in strains lacking all endosomal Rab5s and Ypt7. We thus conclude that CORVET requires only its C-terminal domains for assembly and has beyond its putative β-propeller domains additional binding sites for endosomes, which could be important to bind Vps21 and other endosome-specific factors for efficient endosome tethering.

## Introduction

Endosomes are central organelles, which receive cargo via multiple pathways. Endocytosed material like cell surface receptors arrive from the plasma membrane, whereas biosynthetic cargo such as lysosomal hydrolases travel from the Golgi apparatus to endosomes. On endosomes, decisions are made regarding the fate of the cargo, which can be either sorted to the plasma membrane by recycling routes, or funneled into the endosomal lumen to be then degraded in the lysosome. During their lifetime, the dynamic fission and fusion events change the appearance of endosomes, which mature into multivesicular late endosomes. Along this maturation, endosomes also change their surface composition: whereas early endosomes harbor the small GTPase Rab5, late endosomes are Rab7-positive [1], [2]. Both Rabs are required for endosomal function and interact with multiple effectors that recognize the GTP-loaded Rab. Due to their C-terminal prenyl-anchor, Rab GTPases can cycle between the membrane and the cytosol, where they are bound to the chaperone GDP-dissociation inhibitor (GDI). Recruitment of Rabs seems to require factors that displace the GDI, termed GDP displacement factor (GDF), and allow conversion of the Rab into its active GTP-form [3], [4]. The latter is mediated by GDP/GTP exchange factors (GEFs), and it has been shown for selected GEFs like the bacterial encoded DrrA protein that their GEF activity is sufficient to displace GDI [5].

For fusion, Rabs interact with effectors that bind exclusively to the GTP-loaded Rab [6]–[8]. Endosomes contain at least two characterized Rab effectors that function in fusion, the dimeric early endosomal antigen (EEA1) and similar coiled-coil tethers [9]–[12] and the heterohexameric CORVET complex [13], [14]. Whereas EEA1 seems to have a function restricted to heterotypic fusion of endocytic vesicles with early endosomes [15], [16], CORVET has been implicated in endosome-endosome fusion in yeasts [14], [17], [18]. CORVET, like its sibling, the late endosomal/vacuolar HOPS complex, is a heterohexamer [7], [13], [14], [18], [19]. It shares four subunits with HOPS, namely Vps11, Vps16, Vps18 and Vps33. Vps33 is a Munc18/Sec1-like protein that binds to SNAREs, which reside as the fusion machinery on membranes [20] [21]. The other three subunits Vps11, Vps16 and Vps18 are considered as central templates that allow the interaction with the Rab-specific subunits Vps3 and Vps8 [19], [22]. Interestingly, all HOPS and CORVET subunits with the exception of Vps33 have a similar domain arrangement with a predicted N-terminal β-propeller domain and a C-terminal α-solenoid domain [23]. With the identification of the overall structure of the HOPS complex it became clear that the Rab-specific subunits occupy the ends of the complex and may thus bridge membranes during the fusion process [24]. This model is in agreement with HOPS function in tethering of Rab-decorated membranes [25]. In support of this model, isolated CORVET can efficiently tether vacuole-associated endosomes *in vitro* and *in vivo* [18].

To further understand the function of CORVET, we decided to identify the segments within the Rab-specific subunits that are critical for localization, assembly, and thus function. Here, we report that the predicted N-terminal β-propeller domains of Vps3 and Vps8 are dispensable for CORVET assembly, but not for its function. At least one N-terminal domain is required for efficient endosomal localization and protein sorting. This indicates that complex assembly and function can be assigned to different domains within the CORVET-specific subunits, and that complex integrity does not depend on its localization or functionality. As truncated CORVET, lacking both putative N-terminal β-propeller domains, can still bind to endosomes if the truncated domain is overproduced or if Vps21 amounts are elevated, additional membrane binding sites within the complex must exist. Our data suggest that CORVET depends on the N-terminal domains of the Rab-specific subunits, presumably by binding to Vps21 and other endosome-specific factors during endosomal tethering and fusion.

## Materials and Methods

### Yeast Strains and Molecular Biology

Strains used in this study are listed in Table 1. Tagging of the respective proteins was performed by homologous recombination of the resistance marker together with the tag at the 3′-end of ORF, or together with a promoter at the 5′-end of the ORF [26]. These cassettes were likewise used to truncate Vps3 and Vps8 by replacing indicated parts. Fluorescent tags were as well fused genomically, unless the use of vectors is mentioned. DNA coding for Ste3-GFP was inserted into a pRS425 2 µ based plasmid. Truncated variants of *VPS3* and *VPS8* were amplified together with DNA coding for C-terminal GFP-tags from genomic DNA. Afterwards, they were subcloned into pRS415-*NOP1* promoter *CEN* plasmids using NotI and XhoI restriction sites.

**Table 1:** Strains used in this study.

CUY98	BY4725 *MATa ade2*Δ*::hisG ura3*Δ*0*	Euroscarf™
CUY100	BY4727 *MATalpha his3*Δ*200 leu2*Δ*0 lys2*Δ*0 met15*Δ*0 trp1*Δ*63 ura3*Δ*0*	Euroscarf™
CUY101	BY4728 *MATa his3*Δ*200 trp1*Δ*63 ura3*Δ*0*	Euroscarf™
CUY106	BY4733 *MATalpha his3*Δ*200 leu2*Δ*0 met15*Δ*0 trp1*Δ*63 ura3*Δ*0*	Euroscarf™
CUY473	BY4741 *vps8*Δ*::kanMX*	Euroscarf™
CUY1620	PJ69-4A *leu2 his3 trp1 ade2*	This study
CUY1792	BY4741 *VPS8::TAP-kanMX*	[13]
CUY1797	BY4741 *vps8*Δ*::kanMX VPS3::TAP-URA3*	[13]
CUY2274	BY4741 *VPS8::TAP-URA3*	[34]
CUY2696	BY4733 *VPS21::URA3-PHO5pr-GFP*	[34]
CUY2949	BY4733 *VPS8::3xHA –TRP1 VPS3::TAP-URA3*	[34]
CUY3276	BY4733 *VPS8::GFP -TRP1*	[34]
CUY4191	BY4733 *VPS21::URA3-PHO5pr-GFP VPS8::aa450-TAP-TRP1*	This study
CUY4294	BY4741 *VPS3::kanMX-GAL1pr VPS3::TAP-URA3*	[19]
CUY4352	BY4741 *can1*Δ*::*kanMX	Euroscarf™
CUY4353	BY4741 *art1*Δ*::*kanMX	Euroscarf™
CUY4591	BY4733 *VPS8::aa450-GFP-hphNT1 VPS8::GAL1pr-HIS3*	This study
CUY4595	BY4733 *VPS8::aa450-GFP-hphNT1*	This study
CUY4599	BY4733 *VPS21::URA3-PHO5pr-GFP VPS8::aa450-TAP-TRP1 VPS8::GAL1pr-HIS3*	This study
CUY4640	BY4727 *VPS11::HIS3-GAL1pr VPS16::natNT2-GAL1pr VPS18::kanMX-GAL1pr-3HA VPS33::TRP1-GAL1pr*	[19]
CUY4723	BY4733 *VPS21::URA3-PHO5pr-GFP VPS8::kanMX-GAL1pr*	This study
CUY4771	BY4733 *VPS8::GAL1pr-HIS3*	This study
CUY4852	BY4733 *VPS8::GFP –TRP1 VPS8::HIS3-GAL1pr*	This study
CUY5113	BY4733 *VPS8::yeGFP-TRP1*	[17]
CUY5619	BY4741 *vps3*Δ*::hphNTI*	[17]
CUY5622	BY4733 *VPS8::kanMX-GAL1pr-Vps8-aa451*	This study
CUY5810	BY4733 *VPS8::3xHA –TRP1 VPS3::TAP-URA3 VPS3::NatNT2-CYC1pr-aa427*	This study
CUY5883	BY4733 *VPS8::3xHA –TRP1 VPS3::TAP-URA3 VPS3::NatNT2-CYC1pr-aa427 VPS8::kanMX-CYC1pr-aa451*	This study
CUY6161	BY4741 *VPS8::TAP-URA3 VPS3::-3HA-HIS3*	This study
CUY6540	BY4733 *VPS8::yeGFP-TRP1 VPS3::-3xmCherry-hphNT1*	[13]
CUY6542	BY4741 *VPS8::TAP-URA3 VPS3::-3HA-HIS3 VPS3::kanMX NOP1pr-aa427*	This study
CUY6579	BY4741 *VPS8::TAP-URA3 VPS3::-3HA-HIS3 VPS3::kanMX-NOP1pr-aa427 VPS8::NatNT2-ADH1pr-aa451*	This study
CUY6582	BY4733 *VPS8::3xHA –TRP VPS3::TAP-URA3 VPS8::NatNT2-CYC1pr-aa451*	This study
CUY6703	BY4741 *VPS8::TAP-URA3 VPS3::-3HA-HIS3 VPS3::NatNT2-NOP1pr-aa427 VPS8::kanMX-NOP1pr-aa451*	This study
CUY6705	BY4733 *VPS8::yeGFP-TRP1 VPS3::3xmCherry-hphNT1 VPS3::kanMX-NOP1pr-aa427*	This study
CUY6706	BY4733 *VPS8::yeGFP-TRP1 VPS3::3xmCherry-hphNT1 VPS8::kanMX-NOP1pr-aa451*	This study
CUY6895	BY4733 *VPS8::TAP-URA3 VPS3::3HA-HIS3 VPS8::kanMX-NOP1pr-aa451*	This study
CUY7059	BY4733 *vps3*Δ*::hphNT1 VPS8::TAP-URA3*	This study
CUY7064	BY4741 *VPS8::TAP-URA3 VPS3::3HA-HIS3 VPS8::NatNT2-NOP1pr*	This study
CUY7065	BY4741 *VPS8::TAP-URA3 VPS3::3HA-HIS3 VPS3::kanMX-NOP1pr-aa427 VPS8::NatNT2-NOP1pr*	This study
CUY7066	BY4741 *vps8*Δ*::kanMX VPS3::TAP-URA3 VPS3::NatNT2-NOP1pr-aa427*	This study
CUY7095	BY4733 *VPS3::TAP-kanMX*	This study
CUY7096	BY4733 *VPS3::aa426-TAP-kanMX*	This study
CUY7173	BY4733 *VPS3::aa426-TAP-kanMX VPS3:: NatNT2-GAL1pr*	This study
CUY7286	BY4727/BY4732 (diploid) *VPS11::HIS3-GAL1pr VPS16::NatNT2-GAL1pr VPS18::kanMX-GAL1pr-3HA VPS33::TRP1-GAL1pr/VPS8::TRP1-GAL1pr VPS8::URA3-TAP VPS3::HIS3-GAL1pr VPS3::GFP-hphNT1*	[18]
CUY7564	BY4741 *VPS8::TAP-URA3 VPS3::3HA-HIS3 VPS8::kanMX-NOP1pr-aa451 VPS3::NatNT2-ADH1pr-aa427*	This study
CUY7565	BY4733 *VPS3::TAP-kanMX VPS3::NatNT2-GAL1pr-aa427*	This study
CUY7722	BY4741 *vps8*Δ*::kanMX VPS3::TAP-URA3 VPS3::NatNT2-ADH1pr-aa427*	This study
CUY7723	BY4733 *vps3*Δ*::hphNT1 VPS8::TAP-URA3 VPS8::NatNT2-ADH1pr-aa451*	This study
CUY7983	BY4741 *his3*Δ*1 leu2*Δ*0 met15*Δ*0 ura3*Δ*0 VPS8::TAP-Ura VPS3::-3HA-HIS3 vps21::kanMX*	This study
CUY7946	BY4741 *vps8*Δ*::kanMX VPS3::3HA-hphNT1 VPS3::NatNT2-NOP1pr-aa427*	This study
CUY8378	BY4733 *VPS3::3HA-HIS3 VPS8::aa450-TAP-kanMX*	This study
CUY8379	BY4733 *VPS8::3HA –TRP1*	This study
CUY8383	BY4741 *VPS8::TAP-URA3 VPS3::3HA-HIS3*	This study
CUY8384	BY4741 *VPS8::TAP-URA3 VPS3::3HA-HIS3 VPS3::NatNT2-GAL1pr-aa427 VPS8::hphNT1-GAL1pr-aa451*	This study
CUY8388	BY4741 *his3*Δ*1 leu2*Δ*0 met15*Δ*0 ura3*Δ*0 ypt53::kanMX ypt52::natNT2 vps21::hphNT1 VPS8::-TAP-URA3 ypt7::MET15 VPS3::-3HA-HIS3*	This study
CUY8454	BY4741 *VPS8::TAP-URA3 VPS3::kanMX-NOP1pr-aa427 VPS3::3xmCherry-NatNT2*	This study
CUY8455	BY4741*VPS8::TAP-URA3 VPS3::3xmCherry-NatNT2*	This study
CUY8456	BY4733 *VPS8::yeGFP-TRP1 VPS3::3xmCherry-hphNT1 VPS3::NatNT1-NOP1pr-aa427 VPS8::kanMX-NOP1pr-aa451*	This study
CUY8457	BY4733 *VPS8::yeGFP-TRP1 VPS3::3xmCherry-hphNT1 pRS406-TEF1pr-Vps21 (wt)*	This study
CUY8458	BY4733 *VPS8::yeGFP-TRP1 VPS3::3xmCherry-hphNT1 VPS3::NatNT1-NOP1pr-aa427 pRS406-TEF1pr-Vps21(wt)*	This study
CUY8459	BY4733 *VPS8::yeGFP-TRP1 VPS3::3xmCherry-hphNT1 VPS8::NatNT1-NOP1pr-aa451 pRS406-TEF1pr-Vps21(wt)*	This study
CUY8460	BY4733 *VPS8::yeGFP-TRP1 VPS3::3xmCherry-hphNT1 VPS3::NatNT1-NOP1pr-aa427 VPS8::kanMX-NOP1pr-aa451 pRS406-TEF1pr-Vps21(wt)*	This study
CUY8470	BY4741 *VPS3::3HA-HIS3 VPS8::3xmCherry-NatNT2*	This study
CUY8471	BY4741 *VPS3::3HA-HIS3 VPS8::kanMX-NOP1pr-aa451 VPS8::3xmCherry-NatNT2*	This study
CUY8472	BY4741 *VPS8::TAP-URA3 VPS3::kanMX-NOP1pr-aa427 VPS3::3xmCherry-NatNT2 PEP12::PHO5pr-yeGFP-hphNT1*	This study
CUY8473	BY4741 *VPS8::TAP-URA3 VPS3::3xmCherry-NatNT2 PEP12::hphNT1-PHO5pr-yeGFP*	This study
CUY8474	BY4741 *VPS3::3HA-HIS3 VPS8::3xmCherry-NatNT2 PEP12::hphNT1-PHO5pr-yeGFP*	This study
CUY8475	BY4741 *VPS3::3HA-HIS3 VPS8::kanMX-NOP1pr-aa451 VPS8::3xmCherry-NatNT2 PEP12::hphNT1-PHO5pr-yeGFP*	This study
CUY8568	BY4733 *VPS8::kanMX-GAL1pr-Vps8aa451 VPS8::yeGFP-TRP1*	This study
CUY8589	BY4741 *VPS8::TAP-URA3 VPS3::3HA-HIS3 VPS21::hphNT1-GPD3pr*	This study
CUY8601	BY4741 *VPS8::TAP-URA3 VPS3::3HA-HIS3 VPS3::kanMX-NOP1pr-aa427 VPS8::kanMX-NOP1pr-aa451 VPS21::hphNT1-GPD3pr*	This study
CUY8688	BY4741 *VPS8::TAP-Ura VPS3::NatNT2-GAL1pr-aa427 VPS8::hphNT1-GAL1pr-aa451 VPS3::yeGFP-MET15*	This study
CUY8704	BY4741/BY4727 (diploid) *VPS8::TAP-URA3 VPS3::NatNT2-GAL1pr-aa427 VPS8::hphNT1-GAL1pr-aa451 VPS3::yeGFP-MET15/VPS11::HIS3-GAL1pr VPS16::NatNT2-GAL1pr VPS18::kanMX-GAL1pr-3HA VPS33::TRP1-GAL1pr*	This study

### Microscopy and FM4–64 Staining

Overnight cultures of cells were diluted and further grown to mid logarithmic phase in liquid medium containing 2% glucose. Cells were applied to vacuole staining with the lipophilic dye FM4–64 as described [27]. Alternatively, cells were used for microsopy without staining after being harvested and washed once with synthetic complete medium (SDC) containing all amino acids. Images were acquired using a Leica DM5500 B microscope (Leica, Mannheim, Germany) with a SPOT Pursuit-XS camera (Diagnostic Instruments, Sterling Heights, MI) using filters for GFP, mCherry and FM4–64. All pictures were processed and leveled similarly in Photoshop CS5. Where quantification was necessary, 400 cells were counted randomly.

### Subcellular Fractionation of Yeast Cells

Liquid cultures of yeast cells (20 ml) were treated and lysed as described [27]. Briefly, spheroplasts were generated by lyticase lysis after treatment of cells with DTT. Afterwards, spheroplasts were resuspended in lysis buffer containing 200 mM Sorbitol, 50 mM KOAc, 2 mM EDTA, 20 mM Hepes-KOH, pH 6.8 and protease inhibitors, which was supplemented with DEAE-dextran/HCl to osmotically lyse the spheroplasts during a 2 min heat-shock at 30°C. This cell extract was then cleared by 400 *g* centrifugation for 10 minutes. The supernatant was further centrifuged at 100,000 *g* for 45 minutes to obtain a high-speed pellet containing all organelles of different size and a supernatant containing the cytosolic fraction. Alternatively, the cleared cell extract was used for CPY secretion assays.

### CPY Secretion Assay

Indicated strains were grown in liquid culture to logarithmic growth phase of approximately OD_600_ 1.5–2.5. 20 OD-units of cells were harvested and lysed as described for subcellular fractionation. Lysates were centrifuged for 15 minutes at 13,000 *g* to obtain vacuole-enriched P13 fractions, and proteins were precipitated with 13% trichloroacetic acid (TCA) and loaded onto SDS-PAGE. Western blots were then decorated against CPY and TOM40 as loading control.

### CORVET Purification and Analysis

CORVET was purified via tandem affinity purification (TAP) as described before [13]. In brief, Vps8 was genomically TAP-tagged and isolated from 10,000 OD_600_ equivalents of cells. Lysates were obtained by glass-bead lysis in 50 mM HEPES/KOH, pH 7.4, 300 mM NaCl, 0.15% NP-40 (Igepal CA-630; Sigma-Aldrich), 2 mM MgCl_2_, 1 mM DTT, 1 mM PMSF and 1xFY protease inhibitor mix (Sigma-Aldrich), followed by 100,000 g centrifugation. Clear lysates were applied to 500 µl of pre-washed IgG-beads and incubated for 1.5 h at 4°C. CORVET was eluted from washed beads (20 ml of lysis-buffer, lacking protease inhibitors) by 1 h incubation at 16°C with tobacco etch virus (TEV)-protease. The eluted complex was applied to a linear 10%–40% glycerol gradient, and centrifuged at 35,000 rpm for 18 h in a SW40 rotor at 4°C. One ml fractions were collected, and proteins were TCA-precipitated, loaded onto 7.5% SDS-PAGE, and analyzed by Coomassie-staining. The same protocol was scaled down to analyze protein complexes by western blot [13]. 500 OD_600_ equivalents of cells were used for glass-bead lysis. Lysates were loaded onto 30 µl of pre-washed IgG-beads, which were washed after incubation three times in 1 ml buffer lacking protease inhibitors. Elution was carried out as described above, or alternatively by low pH using Glycin pH 2.5. Eluates were directly TCA-precipitated and analyzed by SDS-PAGE and Western blot. 0.1% load was obtained by TCA-precipitation of total cell extracts of 0.5 OD_600_ equivalents of cells.

### Canavanine Assay

The canavanine assay was done as described [17]. In brief, cells were diluted to OD600 = 0.25 in YPD after growing to mid-logarithmic phase. Based on this, further serial dilutions (1∶5) were done in 5 steps. Cells (2 µl) were then spotted onto synthetic dextrose complete lacking arginine supplemented with indicated amounts of the toxic arginine homolog canavanine. Sensitivity to the drug was assayed by colony-growth after 3–4 days at 30°C.

### GST-Rab Pull-down

Each pull-down reaction was supplemented with 150 µg of recombinant GST-Vps21 or GST-Ypt7 in a total amount of 500 µl Rab loading buffer (20 mM HEPES/NaOH pH7.4, 20 mM EDTA, 150 mM NaCl, 5 mM ß-mercaptoethanol, 7 mg/ml bovine serum albumin and 1 mM GDP or GTPγS, respectively). After an incubation at 30°C for 20 minutes, MgCl_2_ was added to a final concentration of 25 mM. 50 µl of pre-washed GSH-Sepharose (GE Healthcare) was then added to theses samples and incubated for 1 h at 4°C. The loading buffer used for the pre-wash and incubation of the GSH-beads contained 20 mM HEPES/NaOH, pH 7.4, 150 mM NaCl, 5 mM ß-mecaptoethanol, 7 mg/ml bovine serum albumin, 2 mM MgCl_2_ and 0.1% NP-40. The incubation was followed by washing and resuspension of the beads in loading-buffer, which was finally supplemented with 2.5 mM GDP or GTPγS. 35 µg of yeast-purified Vps3-Cbp constructs were loaded onto the beads and incubated for 1 h at 4°C. After incubation, the beads were washed three times in loading buffer without bovine serum albumin, followed by the elution of bound Vps3 constructs at room temperature with the same buffer containing 20 mM EDTA. Eluates were then TCA-precipitated and applied to SDS-PAGE and western blot. The Rabs present in each pull-down were controlled by boiling the beads after elution in SDS sample buffer, followed by SDS-PAGE and coomassie staining.

### Yeast-2-Hybrid Assay

Yeast-two-hybrid assays (Y2Hs) were carried out as described (14). The coding sequences of the bait Vps21 and the Vps8-prey constructs were subcloned into Y2H-vectors (pACT2 and pFBT9). A pair of those vectors was then transformed into the yeast strain PJ69-4A and plated onto synthetic media lacking leucine and tryptophan. After 3 days, transformants were streaked out on these double dropout plates (DDO) to select for both vectors. In addition, the same transfomants were also transferred to triple dropout plates (TDO) and quadruple dropout plates (QDO), which lacked beside leucine and tryptophan also histidine (TDO) and adenine (QDO). Four transformants were analyzed in this way for each combination of bait and prey proteins. A strong interaction between tested proteins resulted in growth on TDO and QDO plates, whereas weak interactions might be indicated by growth on TDO plates only [28].

## Results

### Functional Dissection of the Rab-specific CORVET Subunits

**Figure 1:**
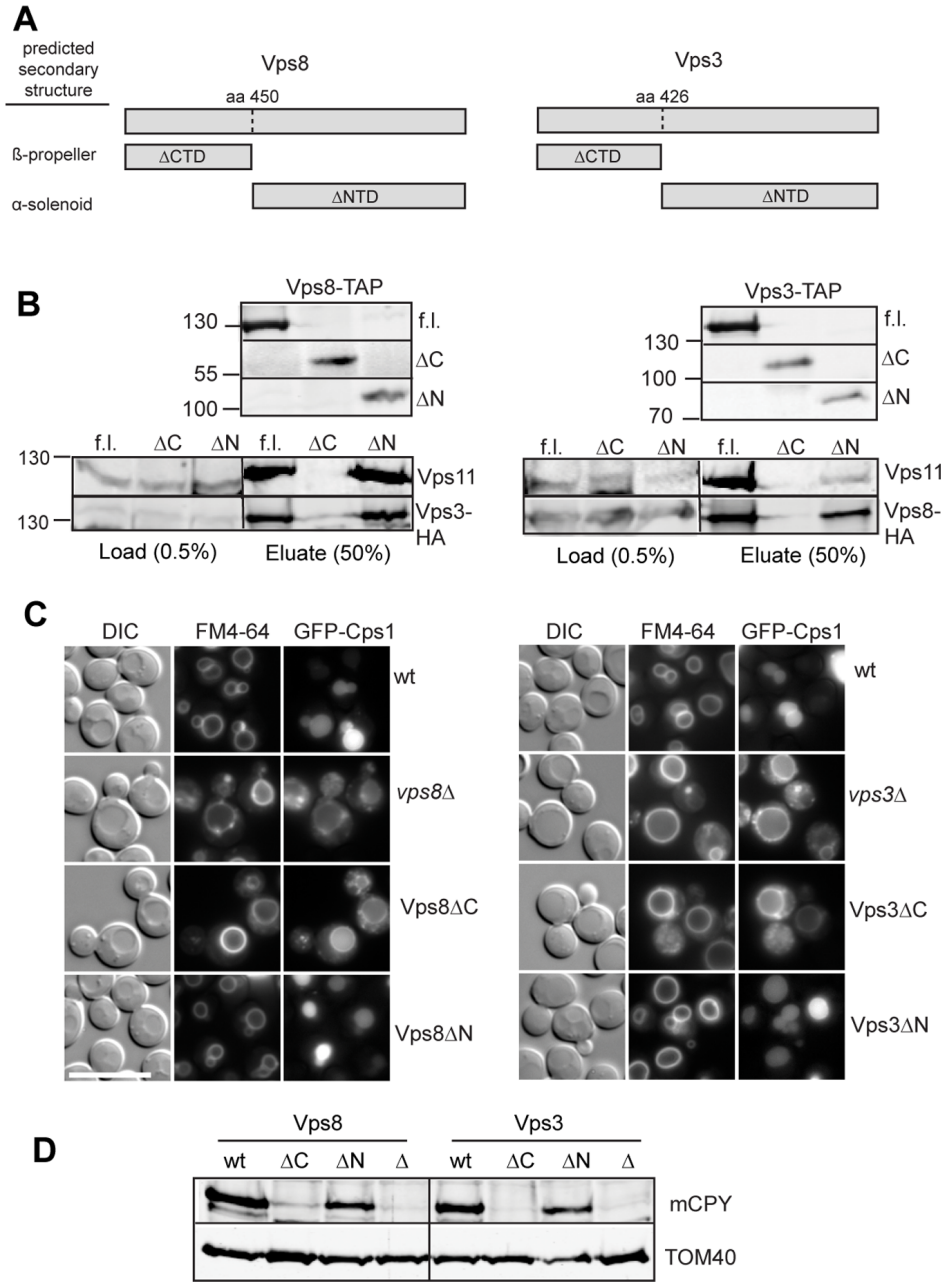
Consequences of domain deletions on CORVET functionality. (A) Domain organization of the CORVET-specific subunits Vps8 and Vps3. N-terminal domains (NTDs/ΔCTDs) correspond to putative β-propeller, whereas C-terminal domains (CTDs/ΔNTDs) represent predicted α-solenoids segments. Domain boundaries were determined by the PredictProtein algorithm (www.predictprotein.org). (B) Purification of truncated Vps3 and Vps8 constructs. Small scale tandem affinity purification (TAP) of Vps8 and Vps3 fragments was carried out via IgG Sepharose. Glycin pH 2.5 - eluates were subjected to SDS-PAGE and Western-Blot via the Odyssey scanning system. Immunoprecipitated CORVET subunits were detected by antibodies against Cbp, Vps11 and HA. Expression of truncated constructs was confirmed by decoration eluates of the pull-down with anti-Cbp antibodies (top panel). Sizes are indicated in kDa. (C) Vacuole morphology and endocytic sorting in Vps3 and Vps8 mutants. Sorting of N-terminally GFP-tagged Cps1, expressed from *CEN*-plasmids, was detected by fluorescence microscopy. To monitor vacuole-morphology, cells were stained with FM4–64. Size bar, 10 µm. See methods for details. (D) Transport of carboxypeptidase Y (CPY). Sorting was monitored by detection of processed CPY (mCPY) from vacuole enriched pellet fractions. Absence of mCPY is due to secretion and defective vacuolar protein sorting. Blots were decorated against Tom40 as loading control.

As the endosomal CORVET and vacuolar HOPS differ only in their two Rab-specific subunits, we decided to dissect the CORVET-specific Vps3 and Vps8 proteins to identify the most critical regions for CORVET function and assembly. Secondary structure prediction programs readily identified a large N-terminal domain in both proteins that likely folds into a β-propeller domain. In addition, both proteins have C-terminal α-helical domains, which could encode α-solenoid domains as predicted by the predictprotein algorithm (www.predictprotein.org) (Figure 1A). We therefore generated truncated versions of both proteins by dividing Vps8 at residue 450 into a construct expressing only the N-terminal domain, termed ΔC or ΔCTD, and another expressing only the C-terminal domain (ΔN, ΔNTD). Vps3 was similarly divided at residue 426. We then tested the expression of the truncated proteins by following the C-terminal tandem affinity purification (TAP) tag. Both N- and C-terminally truncated versions were detected in cells, even though apparently at slightly reduced levels (Figure 1B, top). To analyze if the truncation interferes with complex assembly, we purified Vps3 and Vps8 via their TAP-tags and decorated the eluates with antibodies to Vps11 and the respective other Rab-specific subunit. If either the full-length or N-terminally truncated Vps3 and Vps8 proteins were expressed, we observed the other CORVET subunits in the eluate (Figure 1B). For Vps3ΔN, the complex assembly is potentially slightly reduced. However, this did not affect function, and assembled CORVET could be even purified if both β-propeller domains were missing from Vps3 and Vps8 (see below). In contrast, if the C-terminal domains were lacking, we did not detect any Vps11 or the other Rab-specific CORVET subunit in the pull-down, indicating that complex assembly is defective under these conditions.

To investigate the functionality of the complex, we next asked if protein sorting would be impaired by the truncations. As a first indication, we followed vacuole morphology as vacuoles are significantly larger in *vps3* and *vps8* deletions [17] (Figure 1C). The same enlarged vacuole phenotype was observed in mutants lacking the C-terminal domain of Vps3 or Vps8, which indicates a loss of CORVET function and is consistent with missing CORVET association. In contrast, the vacuoles of mutant cells lacking one of both N-terminal domains appeared like wild-type. To further test this, we followed the sorting of two endosomal cargos to the vacuole. Carboxypeptidase S (Cps1) is a membrane protein that is routed into multivesicular endosomes and further into the vacuole lumen via the ESCRT pathway. Consequently, ESCRT mutants, but also endosomal fusion mutants like *vps3*Δ and *vps8*Δ, accumulate GFP-tagged Cps1 at the vacuolar limiting membrane [17] (Figure 1C). In agreement with the vacuole morphology, mutants expressing Vps8ΔC or Vps3ΔC behaved like the corresponding deletion, whereas mutant cells expressing N-terminally truncated Vps3 or Vps8 had GFP-Cps1 in the vacuole lumen (Figure 1C). As a second cargo, we analyzed the cellular content of soluble carboxypeptidase Y (CPY). In endosomal mutants such as *vps3*Δ and *vps8*Δ, CPY is poorly sorted to the vacuole, and cells have consequently less mature CPY in comparison to wild-type cells (Figure 1D). We also observed low CPY amounts in cells expressing the C-terminally truncated proteins, whereas wild-type cells or those carrying the N-terminally truncated Vps3ΔN or Vps8ΔN contained mature CPY. Overall, our data indicate that the CORVET complex seems to assemble and function in protein sorting if the N-terminal domain of Vps3 or Vps8 is lacking. Furthermore, cells expressing mutants without the C-terminal domain mirror the corresponding deletion, since they are not incorporated into the complex, and we therefore did not analyze them in any further detail.

### Loss of both N-terminal Domains of Vps3 and Vps8 Abolishes CORVET Function

**Figure 2:**
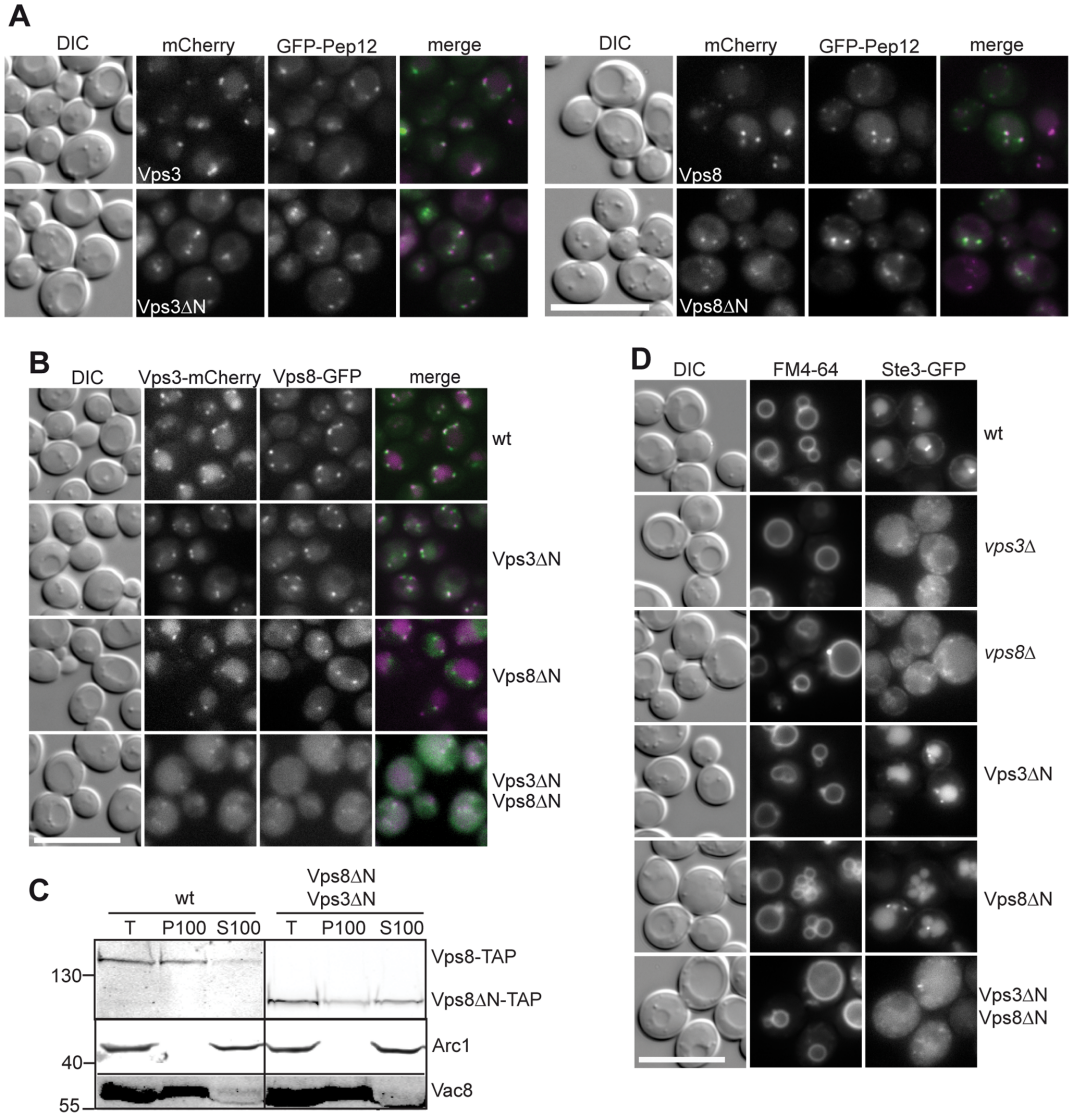
Localization of CORVET requires the N-terminal domains of Vps3 or Vps8. (A) Localization of N-terminally truncated Vps3 and Vps8. Vps3 and Vps8 were genomically tagged with 3xmCherry at their C-temini, and colocalized with genomically tagged GFP-Pep12. Size bar, 10 µm. (B) Colocalization of Vps8 with Vps3. Analysis was performed as in *A*. Vps3 was genomically tagged with 3xmCherry at the C-terminus, whereas Vps8 was tagged with yeGFP. Size bar, 10 µm. (C) Subcellular localization of dually truncated CORVET. TAP-tagged Vps8 was monitored in wild-type and in cells expressing Vps3ΔN and truncated Vps8. Cells (Total, T) were fractionated to obtain a pellet (P100) and supernatant (S100) after the final centrifugation at 100,000 *g*. Western blots were decorated against the TAP tag to identify Vps8. Decoration with antibodies against Arc1 and Vac8 was used as control for cytosolic and membrane-enriched fractions. (D) Endocytosis of Ste3 in cells expressing truncated Vps3 and Vps8. Endocytosis in the respective strains was followed by fluorescence microscopy of C-terminally GFP-tagged Ste3, expressed from 2**µ-plasmids. To monitor vacuole morphology in parallel, cells were treated with FM4–64 beforehand. Size bar, 10 µm.

We then analyzed the role of the Vps3 and Vps8 N-terminal domains by following CORVET localization. In wild-type cells, CORVET is found on endosomal dots as revealed by co-localization with the endosomal SNARE Pep12 (Figure 2A). A similar localization was observed for Vps3ΔN and Vps8ΔN. By co-localization, we further confirmed that these truncated subunits still co-localized with the respective other full-length CORVET subunit, Vps3 or Vps8 (Figure 2B). However, when both N-terminal domains were deleted, the subunits were largely cytosolic (Figure 2B). We could further confirm this, when we fractionated yeast cells. Whereas full-length Vps8 was found on membranes obtained by high-speed centrifugation (P100), truncated Vps8ΔN was largely shifted to the corresponding supernatant. It should be noted that some cellular dots remained nevertheless, which likely correspond to the small portion of membrane-associated Vps8ΔN in the P100 fraction (Figure 2B, bottom, Figure 2C). In agreement with the impaired localization, cells expressing Vps3 and Vps8 without their N-terminal domains were unable to support sorting of the endocytosed Ste3 protein from the plasma membrane to the vacuole lumen (Figure 2D). We thus conclude that the CORVET complex requires at least one N-terminal domain of either Vps8 or Vps3 for its function in endosomal transport.

**Figure 3:**
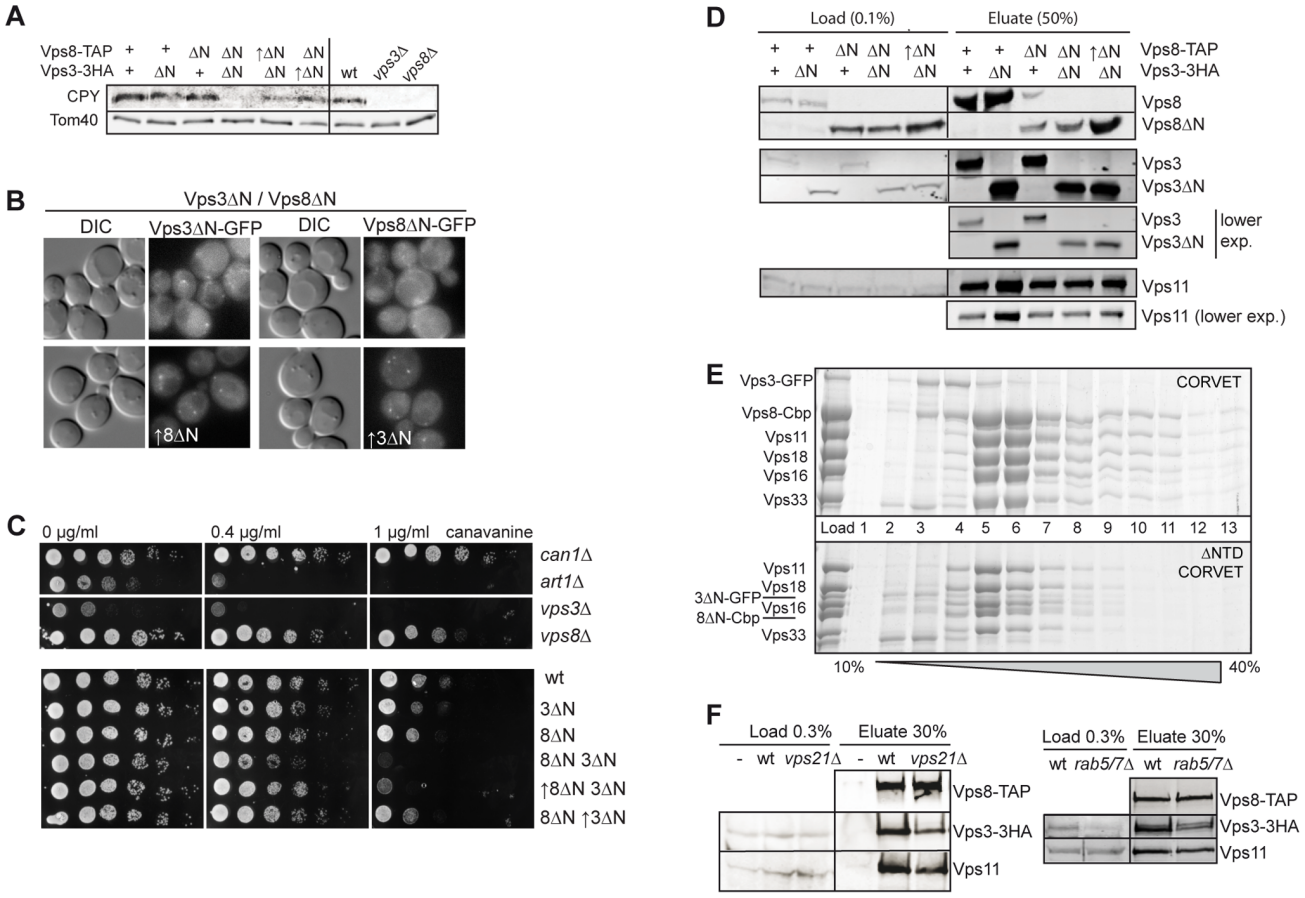
Assembly of CORVET in the absence of the N-terminal domains of Vps3 and Vps8. (A,B) Functional rescue of truncated CORVET. (A) CPY secretion. Membrane fractions were generated from indicated strains carrying TAP- and HA-tagged CORVET subunits. Proteins were resolved on SDS-PAGE gels, and Western blots were decorated using anti-CPY and anti-Tom40 antibodies. Arrows in front of ΔN (deletion of the N-terminal domain) indicate overexpression of the respective constructs, whereas+symbols refer to the presence of full-length proteins. (B) Localization of truncated CORVET. Truncated GFP-tagged Vps3 and Vps8, expressed from *CEN*-plasmids in the respective deletion backgrounds, were followed by fluorescence microscopy in cells where the respective other truncated CORVET subunit was expressed from its endogenous promoter (top) or overexpressed (bottom). (C) Growth sensitivity of truncated CORVET to the toxic arginine homologue canavanine. Strains were grown to logarithmic phase and serial dilutions were spotted onto SDC plates containing indicated concentrations of canavanine [17]. Growth sensitivity is the result of stabilization of the Can1 transporter at the plasma membrane as a consequence of defective endocytosis. (D) Purification of CORVET from mutant cells. Vps8 was C-terminally TAP-tagged and purified using IgG sepharose from wild-type strain and strains expressing truncated versions of CORVET as described in *A*. Proteins bound to the IgG beads were eluted by TEV protease digestion, and resolved by SDS-PAGE. Western blots were decorated with antibodies against the CbP, HA or Vps11. Where indicated, low exposures are shown. (E) Large scale purification of CORVET and ΔNTD-CORVET. Strains carried TAP-tagged Vps8 and GFP-tagged Vps3. Purification was done as described in Methods. The TEV-eluates were applied to 10%–40% glycerol gradients, and centrifuged at 285,000 *g* for 18 h at 4°C. Load indicates 20% of the total eluate. TCA-precipitated proteins of collected 1 ml fractions were loaded onto SDS-PAGE and visualized by coomassie-staining. Cbp, calmodulin-binding peptide that remains on protein after TEV cleavage; 3ΔN-GFP, Vps3ΔN-GFP; 8ΔN-CbP, Vps8ΔN-CbP. The slight band in lane 3 of the eluate that shows Vps8 full-length is due to a slight spill-over from lane 2 as all strains express only the indicated CORVET variant. (F) Purification of CORVET from Rab deletion strains. CORVET was purified via Vps8-TAP from either *vps21*Δ or *vps21*Δ *ypt52*Δ *ypt53*Δ *ypt7*Δ (rab5/7Δ) strains as described for part D. Blots were decorated against the HA-tag on Vps3 and Vps11.

The N-terminal domains are implicated in positioning the CORVET complex on membranes. Importantly, this binding of the CORVET-specific subunits to different membranes is thought to promote tethering. Since CORVET with one truncated Rab-specific subunit is still fully functional in our assays, it is expected, that each subunit has an additional membrane binding site, which is active in tethering, beyond the N-terminal domain. Thus, even the dually truncated complex might function, if this subunit is provided in sufficient quantities. We indeed noticed that some localization of the truncated Vps3 and Vps8 was still observed (Figure 2B). We thus asked if we could compensate for the loss of localization and the subsequent functional deficiencies of CORVET, which lacks both NTDs (ΔNTD-CORVET), by overexpressing one of the two truncated subunits. Overexpression of either Vps3ΔN or Vps8ΔN rescued some CPY sorting (Figure 3A) and dot-localization of the corresponding CORVET partner (Figure 3B). In addition, we followed the sorting of the plasma membrane localized Can1 arginine permease by probing the sensitivity of the respective cells to different concentrations of the toxic arginine analog canavanine. For this, cells were spotted in different dilutions onto plates that were supplemented with indicated concentrations of canavanine and growth was then monitored. Cells lacking Can1 are largely resistant to canavanine, whereas those lacking the Art1 adapter accumulate Can1 on the cell surface and are thus sensitive [29](Figure 3C). We previously showed that cells lacking *vps3* are more sensitive than those lacking *vps8,* whose response to the toxic canavanine is only visible at high concentrations, as shown also here [17]. The sensitivity of those mutants indicates an endocytosis defect for Can1. In agreement with our previous analyses, loss of either N-terminal domain of Vps3 or Vps8 did not affect Can1 trafficking in comparison to wild-type. Even though cells expressing both truncations, Vps3ΔN and Vps8ΔN, show poor localization of these subunits (Figure 2B), they grew better than *vps3*Δ cells (Figure 3C). This already indicates that some function is provided by ΔNTD-CORVET. When we then overexpressed either truncated CORVET subunit, Can1 trafficking was, like the CPY sorting, even partially restored. This indicates that some CORVET function is maintained in the absence of both N-terminal domains, which was enhanced when the truncated subunits were supplied in sufficient cellular concentrations. We then asked if CORVET was indeed still assembled under these conditions. Initially, we monitored the interaction of TAP-tagged Vps8 with Vps3 and Vps11 in cells expressing either the full-length or the truncated versions of CORVET-specific subunits (Figure 3D). As expected, the interaction between Vps8 and Vps3 was maintained regardless of the N-terminal truncations. When Vps8ΔN was overproduced and the interaction with CORVET subunits was monitored, we observed only a slight increase in Vps3ΔN and Vps11 association (Figure 3D, last lane), which can, however, not explain the functional rescue (Figure 3A–C). It is possible that enhanced amounts of truncated CORVET subunits rather stabilize the membrane-association of the complex and thus rescue some function at endosomes.

To compare the composition of the mutant CORVET complex, we decided to overexpress and purify the complex. Complex purification was indeed possible, and the mutant CORVET was detected on glycerol gradients at a comparable molecular weight like the wild-type complex (Figure 3E) [18]. However, the complex could only be purified in the presence of detergent and was thus not suitable for any further functional analyses with membranes. This is in contrast to the entire CORVET complex, which could be purified even in the absence of detergent [18].

As assembly of the CORVET was apparently possible without the N-terminal domains of Vps3 and Vps8, we asked if any endosomal Rab GTPase might be required for its assembly. We thus purified CORVET via Vps8 from either *vps21*Δ cells or from strains lacking all Rab5-like proteins and Ypt7 (*rab5/7*Δ). As shown in Figure 3F, CORVET was still assembled, even though there is no enrichment of CORVET subunits at endosomes under these conditions [17]. We thus conclude that Vps3 and Vps8 do not require an endosomal positioning neither by Rab GTPases nor by their N-terminal domains for efficient complex-assembly.

### Crosstalk of Vps21 with the N-terminal Domains

**Figure 4:**
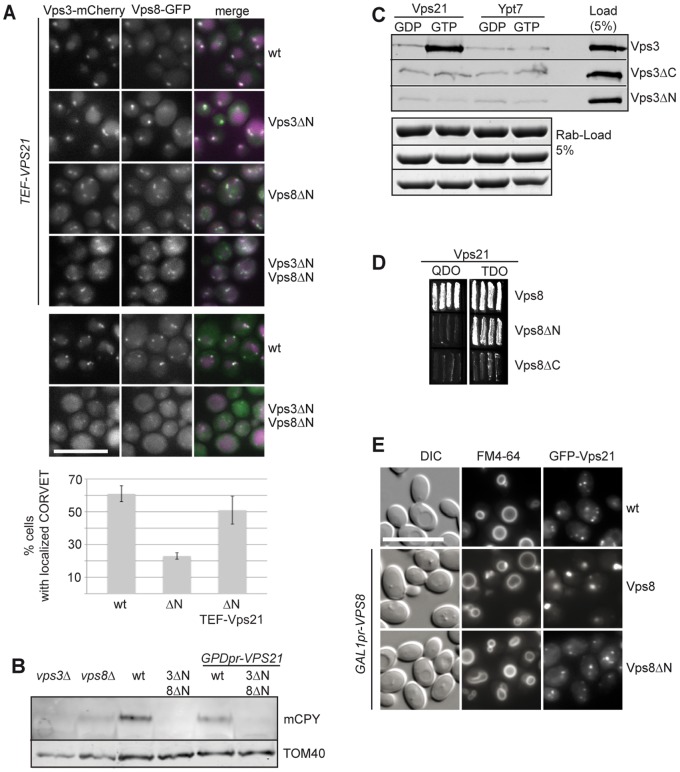
Connection of N-terminal domains of CORVET-specific subunits to the Rab Vps21. (A) Localization of ΔNTD-CORVET upon overproduction of Vps21. TEF1pr-*VPS21* was introduced via integrative pRS406 based plasmids into the indicated background-strains expressing the fluorescently tagged CORVET versions. Cells were monitored by fluorescence microscopy. Statistical analysis of cells with dot-localized CORVET was done by evaluating 400 cells per indicated strain. Cells with multiple dots (at least two) of colocalized Vps3 and Vps8 were counted and given by percentages. Error-bars represent standard deviation of different frames of 50 cells each. Size bar, 10 µm. (B) Functional analysis of Vps21-overexpression in truncated CORVET strains. CPY secretion assay was performed as before (Figure 1D) by isolating membrane fractions, followed by SDS-PAGE, western blot and decorating them against CPY, and TOM40 as loading-control. Vps21 was overproduced by genomically replacing the endogenous Vps21-promoter with the strong *GPD3*-promoter, which is even stronger than the TEF promoter used for our localization assays [26]. (C) Interaction of Vps3 with Vps21. 35 µg of yeast-purified (TAP-purification) Vps3 construct was applied to each pull-down reaction of GSH-beads coupled to GST-Vps21 or GST-Ypt7, which were loaded with GTPγs or GDP beforehand. Eluates were loaded onto SDS-PAGE, followed by western blot, which was decorated against Cbp. SDS-sample buffer was added to beads with bound Rabs afterwards, and proteins were analyzed by SDS-PAGE and Coomassie staining. For details see Methods. (D) Interaction of Vps8 with Vps21. Yeast-2-Hybrid analysis of Vps21-interaction with Vps8 constructs as preys was performed as described in Methods. Growth on QDO (quadruple drop out) plates indicates strong interactions, growth on TDO (triple drop out) refers to weaker interactions [28]. (E) Microscopy images of FM4–64 stained cells overproducing the indicated Vps8 constructs. GFP-Vps21 was used as a fluorescent marker of late endosomal accumulations caused by Vps8 overproduction. Size bar, 10 µm.

CORVET seems to have additional binding sites for factors on endosomal membranes besides those residing in the N-terminal domains of Vps3 and Vps8. Indeed, Vps8 binds in yeast-two-hybrid analyses at regions within the C-terminal segment of the protein to Vps21 [22], [30]. We therefore asked if additional copies of Vps21 could improve the localization and function of ΔNTD-CORVET in mutant cells. Upon overexpression of Vps21, localization of Vps3ΔN and Vps8ΔN to dot-like structures indeed increased significantly (Figure 4A). In contrast, these cells still had a defect in CPY maturation, even though Vps21 expression should be even higher due to the stronger GPD promoter [26](Figure 4B), indicating that Vps21 rescues localization of the mutant CORVET subunits, but not function. It can, however, not be excluded that the additional Vps21 also interferes with endosomal functionality, as hyperactive Vps21 contributes to the accumulation of late endosomes and results in large endosomes in mammalian cells [31]–[34]. To analyze the direct interaction of Vps3 with Vps21, we purified overexpressed full-length Vps3 as well as Vps3ΔN and Vps3ΔC from yeast cells, and incubated these constructs with immobilized Vps21 or Ypt7 that had been precharged with GDP or GTP (Figure 4C). We could detect efficient and specific binding of Vps3 to Vps21-GTP as observed before [22], though both truncations did not show any Vps21 binding *in vitro*. This indicates that that loss of the N-terminal domain of Vps3 affects the interaction with Vps21. Under the same conditions, Vps8 variants were less efficiently purified and bound unspecifically to beads (not shown). We therefore used Vps8 truncations in a yeast-two hybrid assay, and observed an efficient interaction between wild-type Vps8 and Vps21 (Figure 4D). We also detected some binding of Vps8ΔN to Vps21, in agreement with previous studies [22], [30]. This interaction was, however, only observed under less restrictive conditions, indicating a reduced Rab-binding upon loss of the putative β-propeller, similar to the results seen for Vps3. For the N-terminal domain (Vps8ΔC), this assay failed to demonstrate any specific interaction (Figure 4D). We then asked if the N-terminal domain contribute to the interaction with Vps21 *in vivo*. For this, we took advantage of a previous observation, characterized by the ability of Vps8 to drive Vps21 into dot-like structures, which correspond to accumulated MVBs [34](Figure 4E). When we performed the same assay with N-terminally truncated Vps8, Vps21 was found in multiple endosomal dots, which reflects its localization in wild-type cells (Figure 4E) [17]. Our data thus indicate that the Vps8 N-terminal domain contributes to the interaction with Vps21, which could be important to allow for efficient tethering at endosomal membranes.

## Discussion

Within this study, we aimed to dissect the domains of the Rab-specific subunits Vps3 and Vp8 of the endosomal CORVET complex. We could reveal that the C-terminal α-helical segments of both proteins are necessary for CORVET assembly and function, as mutants lacking these parts behaved like deletions of the entire protein (Figure 1). More informative was the further analysis of mutants expressing N-terminally truncated Vps3 and Vps8. Interestingly, cells behaved like wild-type in terms of complex assembly, endocytic protein sorting and localization, if only one N-terminal domain was lacking (Figure 1, 2). If both putative β-propeller were deleted, the mutant CORVET complex could still assemble, but localized poorly and was functionally strongly impaired (Figure 2). Furthermore, assembly required neither endosomal Rab5-like proteins nor the vacuolar Ypt7, which highlights the ability of the complex to assemble in the cytoplasm solely dependent on its C-terminal domains. The deficiencies of ΔNTD-CORVET in localization and endosomal sorting could be rescued by providing additional copies of the truncated subunits. Thus, the N-terminal domains are not the only determinants of CORVET for endosomal positioning. Instead, additional membrane-binding domains exist within the complex and likely within the C-terminal parts of Vps3 and Vps8. Our data further show that Vps21 is one limiting factor that determines localization of the truncated CORVET, in support of additional Vps21 binding sites within the C-terminal part of Vps8 [22], [30] (Figure 4). As overexpression of Vps21 may affect endosomal transport itself [33], we cannot yet say if the defective endosomal sorting observed under these conditions is due to missing functions in CORVET or to Vps21-overproduction.

The results obtained here are largely consistent with previous findings on the homologous HOPS complex. For HOPS, we identified Rab-binding to the opposite ends of the complex, and could reveal interactions between Ypt7-GTP and the N-terminal domain of Vps41 or full-length Vps39 [19], [22]. Vps39 and Vps3 both interact with the shared Vps11 subunit via their C-terminal segments, which explains the critical role of this part for complex assembly [19], [22]. Likewise, neither Vps41 [35] nor Vps8 (as shown here) seem to function if their C-terminal segments have been removed. This similarity indicates that HOPS and CORVET depend on the same segments for their integration into the tethering complex.

For efficient membrane tethering, CORVET seems to require at least one of its N-terminal domains in the Rab-specific subunits. Apparently, these domains are required for a binding to endosome-specific factors such as Vps21, phosphoinositide-3-phosphate or selected SNAREs. As the isolated N-terminal domains behaved poorly in our hands, we could not yet reveal if they directly interact with any of these. At least *in vitro*, full-length CORVET recognizes Vps21-GTP efficiently, and requires this interaction to tether vacuole-associated endosomes *in vitro* and *in vivo* [18]. As the dually truncated complex (ΔNTD-CORVET) disassembled during our biochemical analysis, we could not test its binding to Vps21-GTP or endosomal lipids. A detailed analysis of the N-terminal domains was further hampered by the poor behavior of the isolated fragments (not shown). In addition, CORVET has apparently additional membrane binding sites that do not reside in the analyzed N-terminal domains of Vps3 and Vps8. For Vps8, Vps21-binding sites have been identified also along the C-terminal part, and can thus explain the wild-type like behavior of cells with Vps8ΔNTD [22], [30]. For Vps3, only the N-terminal domain has been demonstrated as the Vps21-binding site [22], which would thus be deleted in the ΔNTD-CORVET mutant. We therefore postulate that Vps3 also binds membranes via additional surfaces along the C-terminal domain. The binding of CORVET via multiple endosome-specific factors would ensure its specific localization and function in endosomal tethering. To understand the precise contribution of each N-terminal domain of Vps3 and Vps8, future assays will need to precisely map the membrane binding sites along the complex to understand their contribution to tethering and fusion.
